# Theoretical derivation and experimental investigation of dynamic displacement reconstruction based on data fusion for beam structures

**DOI:** 10.1038/s41598-022-24449-2

**Published:** 2022-11-19

**Authors:** Liang Ren, Qing Zhang, Xing Fu

**Affiliations:** State Key Laboratory of Coastal and Offshore Engineering, Dalian University of Technology, Dalian, 116023 China

**Keywords:** Civil engineering, Mechanical engineering

## Abstract

Accurately obtaining the dynamic displacement response of the beam structure is of great significance. However, it is difficult to directly measure the dynamic displacement for large structures due to the low measurement accuracy or the installation difficulty of the sensor. Therefore, it is urgent to develop an indirect measurement method for displacement based on measurable physical quantities. Since acceleration and strain contain high and low frequency displacement information respectively, this paper proposes a displacement reconstruction algorithm that can realize the data fusion of the two, which is very helpful for the research of structural health monitoring. Firstly, the stochastic subspace identification (SSI) method is adopted to calculate the strain mode, and then the displacement is derived via the mode shape superposition method. Afterwards, the strain-derived displacement and acceleration are combined by the proposed algorithm to reconstruct the dynamic displacement. Both the numerical simulation and model experiment are conducted to verify the effectiveness of the proposed algorithm. Furthermore, the influences of noise, sampling rate ratio and measurement point position are analyzed. The results show that the proposed algorithm can accurately reconstruct both high-frequency and pseudo-static displacements, and the displacement reconstructed error in the model experiment is within 5%.

## Introduction

Structural health monitoring is an important guarantee for the long-term operation of the structure^1–3^, whose purpose is to evaluate the health of the structure and to give a reference to whether the structure needs maintenance, which plays a vital role in the safe operation of large-scale infrastructure during service. Dynamic displacement is one of the key parameters for evaluating the safety performance of structure^4^, which is widely used in the field of structural health monitoring and vibration control owing to provide information directly related to structural deformation and flexibility^5–7^. Nevertheless, it is difficult to measure dynamic displacement of actual structures directly. On the one hand, contact displacement sensors such as linear variable differential transformer (LVDT) requires a fixed reference point, but the reference point tends to move due to various reasons. On the other hand, non-contact sensors like GPS still have various issues, such as low sampling rates and poor accuracy that have not been solved. As a result, research on structural dynamic displacement reconstruction has received extensive attention^8,9^.

In the past few decades, scholars have done a lot of work on the reconstruction of dynamic displacement^10,11^. A single type data easy to measure was used to achieve displacement reconstruction at the beginning^12–14^. For example, Lee et al.^15^ designed a finite impulse response (FIR) filter to instruct the acceleration integration process, and effectively reduced the acceleration integration error. However, the selection criteria of the optimal regularization factor in this method has not been clearly deterministic and is only applicable to zero mean dynamic displacement reconstruction. Shin et al.^16^ applied the theoretical displacement mode shape of the simply supported beam and the strain measured by the fiber grating sensor to estimate displacement. Nevertheless, it is unrealistic for the displacement mode to completely match the theoretical one for the actual bridge structure. Wang et al.^17^ used the strain response to derive the strain mode, then calculated the displacement mode, and finally employed the mode superposition method to obtain the dynamic displacement of a simply supported beam. While this method requires a higher sampling rate, and the existing health monitoring system is difficult to meet this requirement. Obviously, there are many limitations to achieve displacement reconstruction by using a single type data.

To utilize the redundant information of multiple types of sensors as much as possible and to improve the accuracy of dynamic displacement reconstruction, the displacement reconstruction method based on multiple types data has been studied^18–20^. Roberts et al.^21^ applied GPS measurement data and its derivative to correct the integral process of acceleration, realized the data fusion of GPS measurement data and acceleration. Even so, the existence of signal measurement noise makes the integration and differentiation of measurement data inaccurate. The disadvantages of low accuracy, expensive equipment and low sampling rate of GPS also limit the application of the above methods in civil engineering. Park et al.^22^ applied strain-derived displacement to guide the acceleration double integration process, and successfully achieved non-zero mean dynamic displacement reconstruction. Cho et al.^23^ used the theoretical strain mode shape to derive the displacement from the strain, and then utilized the Kalman filtering algorithm to incorporate the acceleration. The same rate data is used in the aforementioned data fusion algorithms, while most of the collected multiple types data are in a state where multiple rates coexist in the current health monitoring system. And the modal information of the actual structure may be different from the theoretical value, and the operation modal analysis method should be used for modal identification.

For the purpose of solving the problem of multi-rate data information fusion, researchers have introduced Kalman filtering technology into dynamic displacement reconstruction^24–26^. Smyth et al.^27^ suggested the use of Kalman filtering technology to perform multi-rate data fusion of displacement and measured acceleration with different sampling rates, and the effectiveness of the method was proved by numerical simulation. Kim et al.^28^ estimated the dynamic displacement in real-time by fusing the measured velocity from the laser Doppler vibrometer (LDV) along with the measured displacement from the light detection and ranging (LiDAR), which improved the poor accuracy and low sampling rate of the LiDAR, finally obtained high sampling rate and precision dynamic displacement. It is worth noting that although the above Kalman filtering algorithms solved the problem of multi-rate data fusion, the usage of physical quantities that are difficult to measure in actual engineering (such as displacement or velocity), limits its practical application.

The above research shows that many scholars have done a lot of exploratory work in dynamic displacement reconstruction, as well as provide a variety of methods to achieve it^29^. The existing data fusion algorithms have many limitations, such as depending on the theoretical mode shapes of the structure, unable to deal with the inconsistency between the acceleration and the strain sampling rate, and requiring more measurement points. Therefore, this paper proposes a multi-rate data fusion algorithm that relies on the measured data to realize mode parameters identification and displacement reconstruction, which breaks through the above limitations. The proposed algorithm distinguishes itself from the existing methods by utilizing a novel mode shape superposition method and fuse strain-derived displacement with high sample rate acceleration, which improves both the accuracy and convenience of displacement reconstruction. Compared with the previous research^30^, the method proposed in this paper explicitly considers the error of the acceleration sensor, and the acceleration integral error and displacement error are considered as state variables to further improve the reconstruction accuracy. Moreover, the research object of this paper is cantilever structure while previous study focused on simply supported structure. In “Displacement reconstruction method from multi-rate data”, the theory of above-mentioned improved multi-rate Kalman filtering algorithm is introduced. In “Case study using a cantilever beam”, a cantilever beam finite element model is established for numerical simulation verification, and we compared with existing methods, including different mode identification and displacement reconstruction methods. Afterwards, parameter analysis is carried out. In “Experimental validation”, the vibration experiment and parameter analysis of the cantilever beam model are performed and compared with the simulation results in “Case study using a cantilever beam”. Finally, “Summary and conclusion” summarizes the full text and gives a conclusion.

## Displacement reconstruction method from multi-rate data

To solve the problem of information fusion of data with multiple types and rates in existing research, an improved multi-rate data displacement reconstruction method is proposed. Different from the existing multi-rate Kalman filtering algorithm based on acceleration and displacement^31^, the proposed improved multi-rate Kalman filtering algorithm fuses the displacement derived from strain with the measured acceleration to estimate the acceleration integration error, and finally realizes dynamic displacement reconstruction. This section introduces the mode superposition method and the multi-rate Kalman filtering algorithm respectively.

### Strain mode superposition method for beam structure

The derivation process of the mode superposition method based on the SSI algorithm can refer to the existing literature, and the important parts are introduced here^32^. Dynamic displacement $u(x,t)$ and strain response $\varepsilon (x,t)$ have the same modal coordinates, and both of them can be expressed as the superposition of the first *n*-order modes:

1 $$ u(x,t) = \sum\limits_{i = 1}^{n} {\Phi_{i} (x)q_{i} (t)} $$

2 $$ \varepsilon (x,t) = \sum\limits_{i = 1}^{n} {\Psi_{i} (x)q_{i} (t)} $$

where $\Phi_{i} (x)$ denotes the modal displacement of the *i*th-order displacement mode shape; $q_{i} (t)$ is the *i*th-order modal coordinate; *n* represents the modal order used; and $\Psi_{i} (x)$ represents the modal displacement of the *i*th-order strain mode shape.

The SSI method based on data-driven is more applicable for the actual structure under long-term environmental load^33,34^. The strain mode shapes of the structure can be ultimately obtained as follows via the identification of the system matrix ${\mathbf{A}}_{c}^{\varepsilon }$ and ${\mathbf{C}}_{c}^{\varepsilon }$:

3 $$ {\mathbf{A}}_{c}^{\varepsilon } = \chi {{\varvec{\Lambda}}}\chi^{ - 1} $$

4 $$ \Psi = {\mathbf{C}}_{c}^{\varepsilon } \psi $$

where $\Psi$ represents the strain mode of the structure; $\chi$ is the eigenvector; ${{\varvec{\Lambda}}}$ is the diagonal eigenvalue matrix. Then the modal conversion method is used to convert the identified strain mode into displacement mode, there are the following formulas for cantilever beam structures according to the beam bending theory in material mechanics:

5 $$ u(x)^{\prime\prime} \cdot d = d \cdot \frac{M(x)}{{EI}} = \varepsilon (x) $$

where *d* represents the distance from the measurement point to the neutral layer of the structure. The position of the neutral layer can be determined by parallel strain experiments; $\varepsilon (x)$ means the strain, $M(x)$ represents the bending moment; *EI* stands for the bending stiffness of the beam and $u(x)$ represents the displacement.

Extending Eq. (5) to a period of time, we can get:

6 $$ \varepsilon (x,t) = d \cdot \frac{{\partial^{2} u(x,t)}}{{\partial x^{2} }} = d \cdot \sum\limits_{i = 1}^{n} {\Phi^{\prime\prime}_{i} (x)q_{i} (t)} $$

Integrating the left and right ends twice at the same time to get the *i*th-order displacement mode function:

7 $$ \Phi_{i} (x) = \frac{1}{d} \cdot (\iint {\Psi_{i} (x)dx^{2} ) + Cx + D} $$

where *C* and *D* are two integral constants, both of them are 0 for cantilever beam.

After calculating the first *n*-order displacement mode shapes, composing them into a displacement mode matrix. The displacement response of the structure $u(x,t)$, denoted as $x_{strain}$, can be obtained by substituting the calculated strain mode matrix $\Psi_{i} (x)$ and displacement mode matrix ${{\varvec{\Phi}}}(x)$ into Eqs. (1) and (2).

### Improved multi-rate Kalman filtering algorithm

Different from existing studies, the improvement of the proposed method is that the observed value used is the strain-derived displacement. Therefore, only the general process of the Kalman filter algorithm is introduced here. For details, please refer to the existing literature. The state variables of the continuous-time state space model of Kalman filtering are selected as:

8 $$ {\mathbf{X(}}t{\mathbf{)}} = \left[ {\begin{array}{*{20}c} {\lambda x(t)} & {\lambda \dot{x}(t)} & {a(t)} \\ \end{array} } \right]^{T} $$

where $\lambda x(t)$ is the displacement error; $\lambda \dot{x}(t)$ means the speed error. The discrete form is:

9 $$ {\mathbf{X}}(k) = {{\varvec{\Phi}}}(k\left| {k - 1} \right.){\mathbf{X}}(k - 1) + {{\varvec{\Gamma}}}(k\left| {k - 1} \right.)w(k - 1) $$

where ${{\varvec{\Phi}}}(k\left| {k - 1} \right.)$ represents the state transition matrix; ${{\varvec{\Gamma}}}(k\left| {k - 1} \right.)$ denotes the noise process vector and

10 $$ {{\varvec{\Phi}}}(k\left| {k - 1} \right.) = \left[ {\begin{array}{*{20}c} 1 & {\Delta t} & {0.5\Delta t^{2} } \\ 0 & 1 & {\Delta t} \\ 0 & 0 & 1 \\ \end{array} } \right]{, }{{\varvec{\Gamma}}}(k\left| {k - 1} \right.) = \left[ {\begin{array}{*{20}c} {0.5\Delta t^{2} } \\ 0 \\ 0 \\ \end{array} } \right] $$

The covariance matrix of one-step state prediction can be expressed as:

11 $$ {\mathbf{P}}(k\left| {k - 1} \right.) = {{\varvec{\Phi}}}(k\left| {k - 1} \right.){\mathbf{P}}(k - 1\left| {k - 1} \right.){{\varvec{\Phi}}}(k\left| {k - 1} \right.)^{T} + {\mathbf{Q}} $$

where ${\mathbf{P}}(k - 1\left| {k - 1} \right.)$ denotes the covariance matrix; ${\mathbf{Q}}$ denotes the stochastic noise covariance matrix:

12 $$ {\mathbf{Q}} = q\left[ {\begin{array}{*{20}c} {0.25\Delta t^{4} } & {0.5\Delta t^{3} } & 0 \\ {0.5\Delta t^{3} } & {\Delta t^{2} } & 0 \\ 0 & 0 & 0 \\ \end{array} } \right] $$

Supposing that the strain-derived displacement is obtained at the time step *i*, the displacement error at the time step *i* can be updated:

13 $$ y(i) = \hat{x}(i) - x_{strain} (i) $$

where $y(i)$ is the calculated displacement error. The displacement error can also be obtained from the observation process:

14 $$ {\mathbf{Z}}(i) = {\mathbf{HX}}(i\left| i \right. - 1) + v(i) $$

where ${\mathbf{Z}}(i)$ denotes the observed displacement error; the observation matrix $${\mathbf{H}} = \left[ {\begin{array}{*{20}c} 1 & 0 & 0 \\ \end{array} } \right]$$; $v(i)$ means the uncertainty associated with the observation process, assuming that this uncertainty obeys a stationary zero mean Gaussian process, namely $v(i) \sim N(0,r)$. Thus, the state estimation vector ${\mathbf{X}}(i\left| i \right.)$ and state estimation covariance matrix ${\mathbf{P}}(i\left| i \right.)$ in the measurement update process are:

15 $$ {\mathbf{X}}(i\left| i \right.) = {\mathbf{X}}(i\left| {i - 1} \right.) + {\mathbf{K}}(i)(y(i) - {\mathbf{HX}}(i\left| {i - 1} \right.)) $$

16 $$ {\mathbf{P}}(i\left| i \right.) = ({\mathbf{I}} - {\mathbf{K}}(i){\mathbf{H}}){\mathbf{P}}(i\left| {i - 1} \right.) $$

where **I** denotes the unit matrix; ${\mathbf{K}}(i)$ represents the Kalman gain matrix at the time step *i*, calculated by the following formula:

17 $$ {\mathbf{K}}(i) = {\mathbf{P}}(i\left| {i - 1} \right.){\mathbf{H}}^{T} ({\mathbf{HP}}(i\left| {i - 1} \right.){\mathbf{H}}^{T} + r)^{ - 1} $$

where *r* indicates the variance of the measurement noise during the observation process.

According to the state estimation vector acquired from the measurement update process, the acceleration integral results can be further corrected:

18 $$ \hat{\dot{x}}(i\left| i \right.) = \dot{x}(i) - \lambda \dot{x}(i\left| i \right.) $$

19 $$ \hat{x}(i\left| i \right.) = x(i) - \lambda x(i\left| i \right.) $$

where $\hat{x}(i\left| i \right.)$ represents the reconstructed displacement; $\hat{\dot{x}}(i\left| i \right.)$ means the reconstructed speed.

**Figure 1:**
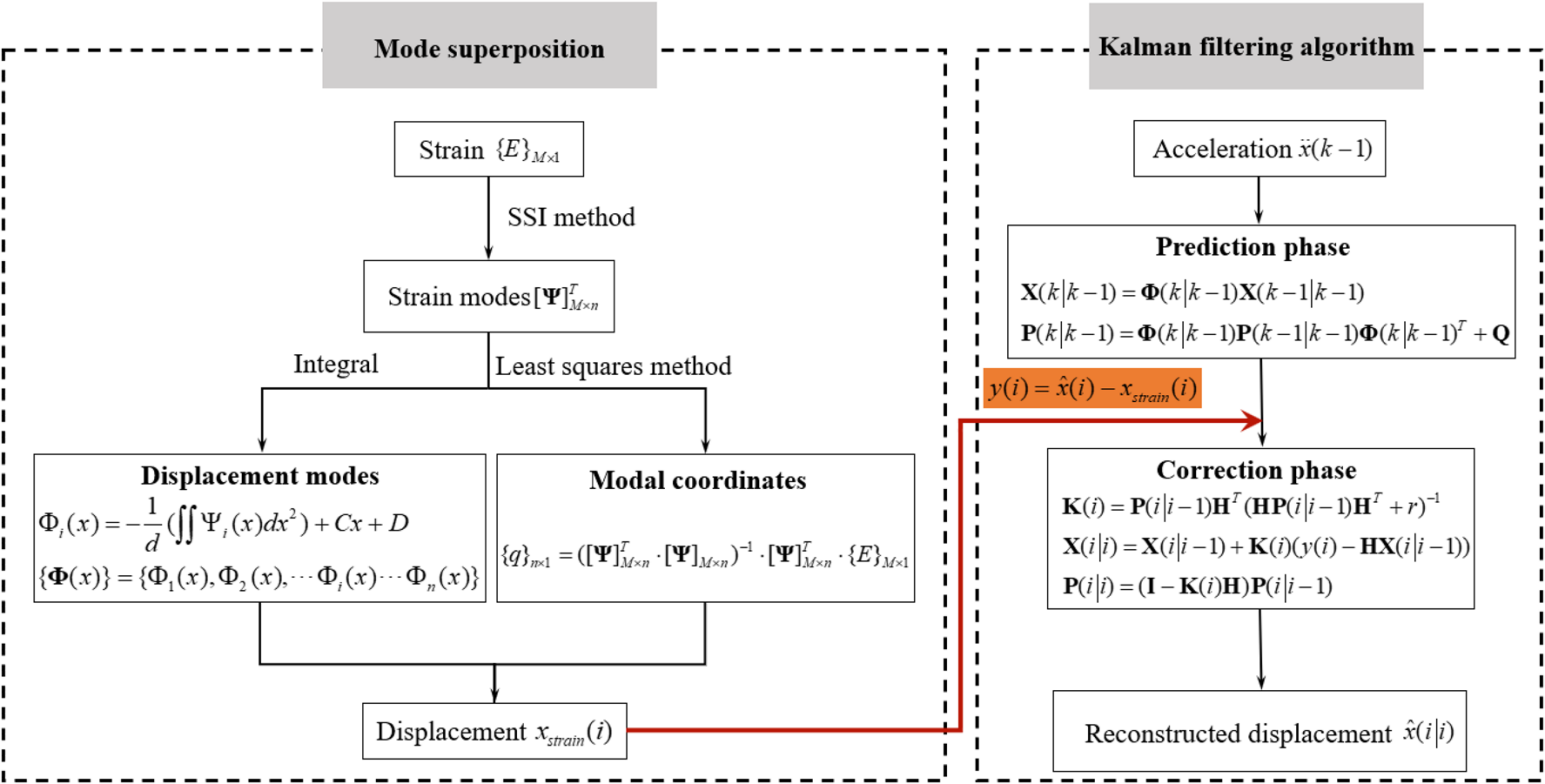
Flow chart of multi-rate data displacement reconstruction method.

The flow chart of the proposed multi-rate data displacement reconstruction method is shown in Fig. 1. Only the important steps are introduced here for the sake of brevity, corresponding details and smoothing technology could refer to Kim et al.^31^.

## Case study using a cantilever beam

### Simulation scenario

To verify the effectiveness of the above-mentioned multi-rate data fusion algorithm, the ANSYS software was used to establish the three-dimensional finite element model of a cantilever beam. A steel beam of $800{\text{ mm}} \times 30{\text{ mm}} \times 3.75{\text{ mm}}$ was simulated by BEAM188, the material constants of the beam are Elastic modulus = 206 GPa and Poisson's ratio = 0.3.

**Figure 2:**
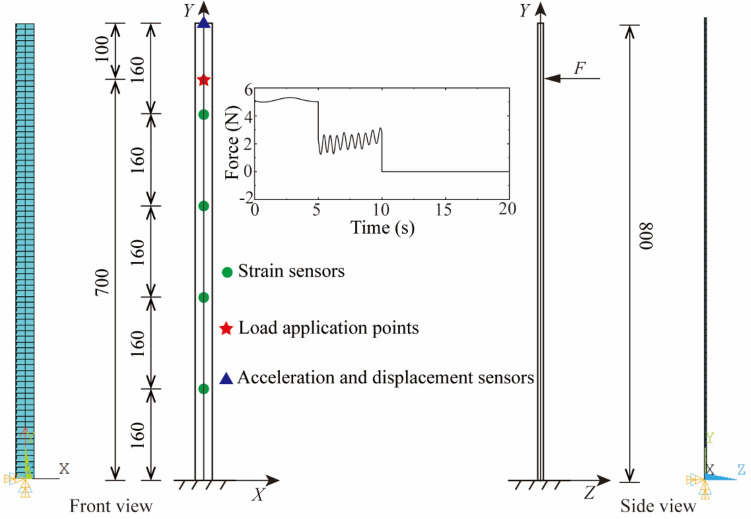
Finite element model and measurement point layout drawing (unit: mm).

Considering that the vibration of the first three-order mode shapes is dominant when the structure vibrates, and the number of measurement points needs to be greater than the order of mode shape to identify the mode shape, four strain sensors were arranged on the entire model evenly from top to bottom, the free end endpoint was set as the acceleration and displacement sensors. The cantilever beam model was excited along the *Z* direction for a duration of 20 s, then the acceleration and strain response sampling rate were set to 1200 Hz and 300 Hz respectively. The finite element model and measurement point layout are shown in Fig. 2.

### Displacement reconstruction

**Figure 3:**
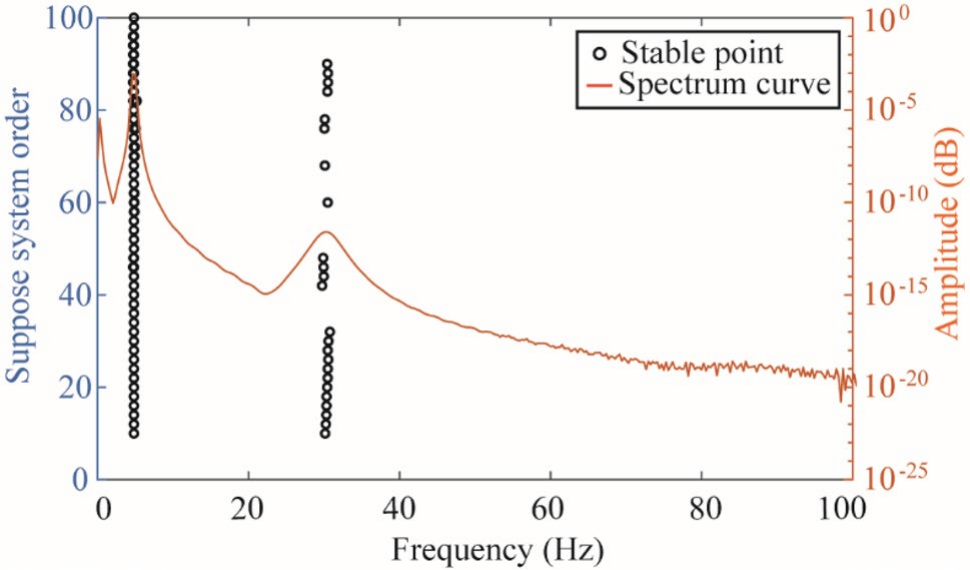
Stability diagram and spectrum diagram.

The strain response time history extracted by ANSYS software was used to identify the strain mode via SSI method, and the stability diagram and spectrum diagram were shown in Fig. 3. The circles in the stability diagram represent the points where the modal parameters have stable solutions and the orange curve represents the spectrum curve.

**Figure 4:**
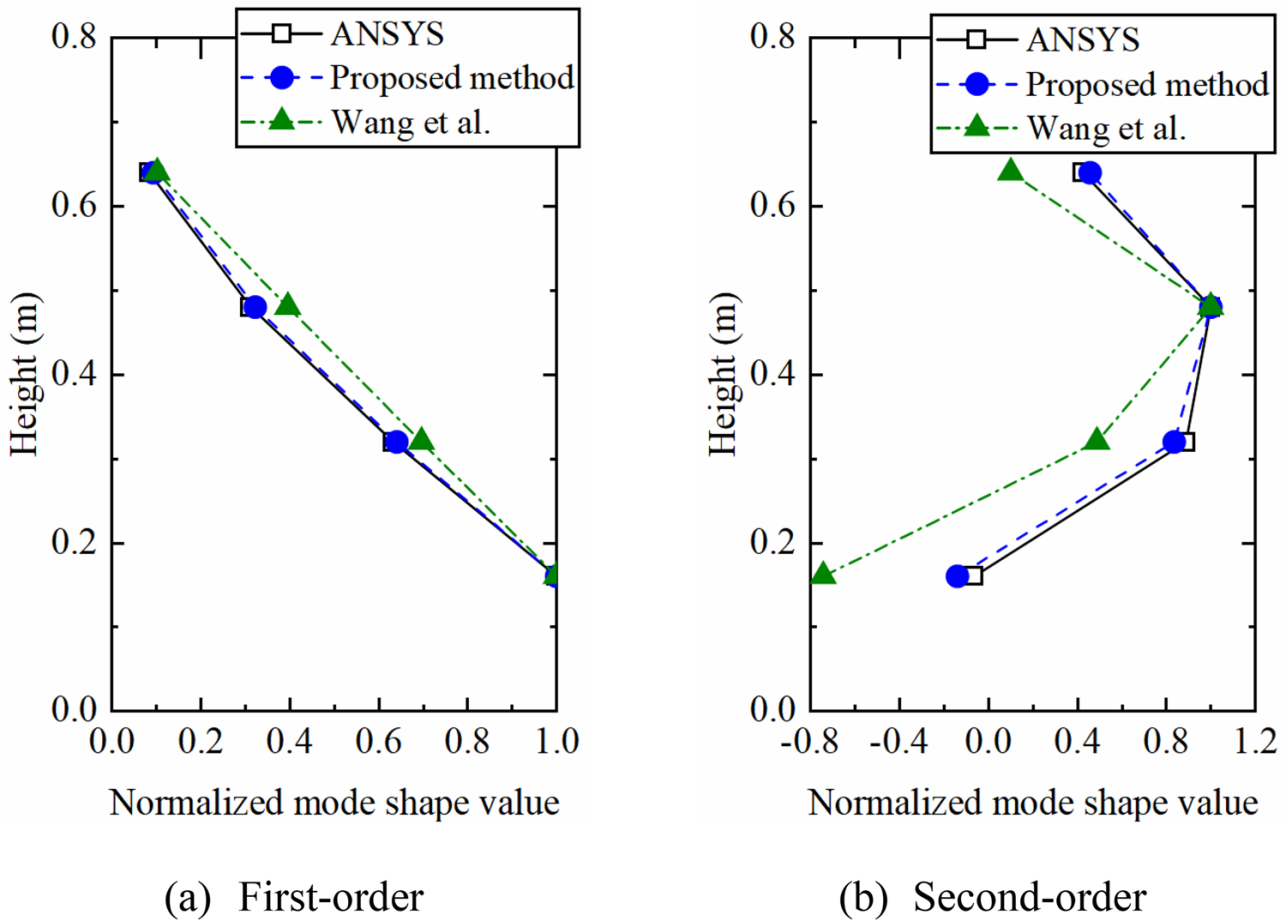
Strain mode shapes calculated by different methods.

Considering the stable axis and the spectrogram comprehensively, the first two-order strain modes occupy most of the vibration energy clearly, so only the first two-order strain modes are extracted, and they are compared with the strain mode shapes extracted by ANSYS and that calculated by the method proposed by Wang et al.^17^ as shown in Fig. 4. The calculated results of proposed method are closer to the theoretical value whether the first-order or second-order mode, indicating the superiority of the proposed method.

**Figure 5:**
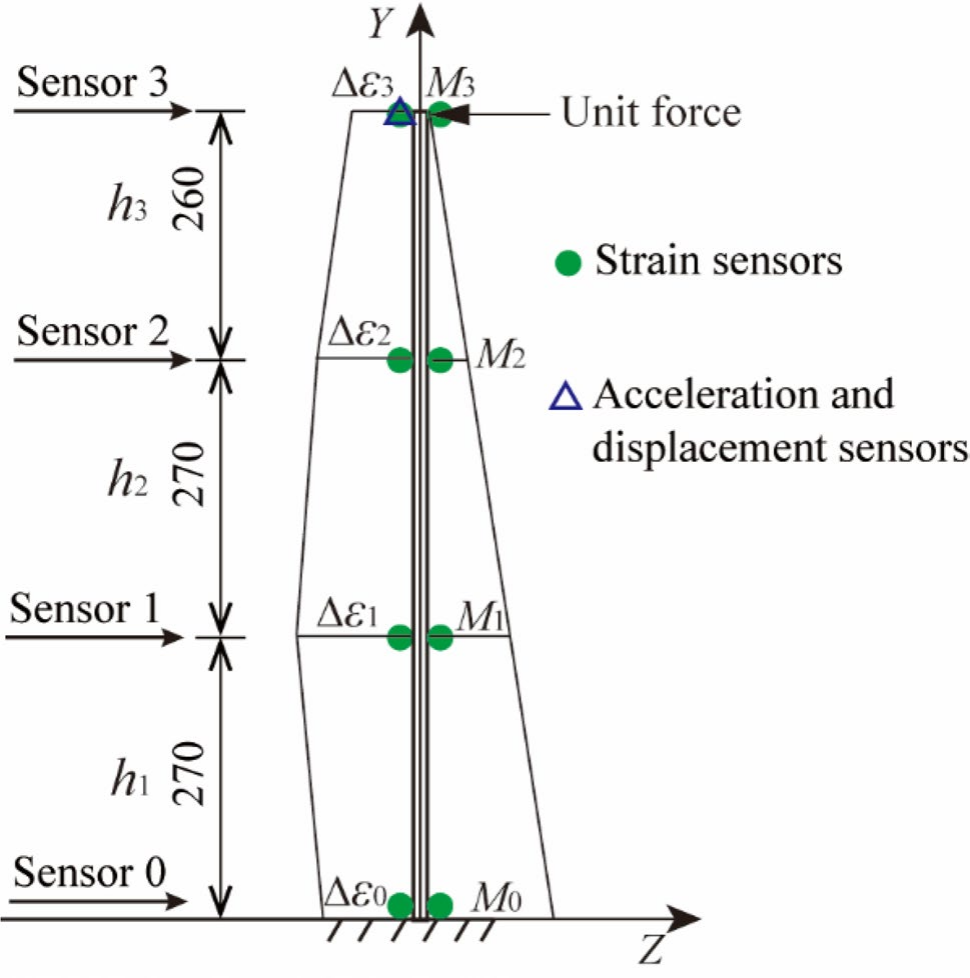
Schematic diagram of measurement points layout and parameters (unit: mm).

According to the mode superposition method, the displacement mode can be calculated from the strain mode and the low sampling rate dynamic displacement was obtained, then the proposed multi-rate Kalman filtering algorithm was used to reconstruct the displacement. In addition, the displacement reconstruction method proposed by Zhu et al.^26^ was used for comparison, the measurement points required by this method are 0 m, 0.27 m, 0.54 m and 0.8 m away from the fixed end. The strain data on both the left and right sides for each measurement point are needed to be extracted to calculate the strain difference, which means there are eight strain sensors. The method has established the mapping relationship between strain difference on both sides and displacement of the same measuring point, so the strain sensor must be arranged at the target measuring point. Nevertheless, the method proposed in this paper fits the function relation between the mode shape value and the corresponding height value. Even if the strain sensor is not installed at the target point, the height of the target can be substituted into the mode shape function to obtain the mode shape value, and then the dynamic displacement can be obtained. The schematic diagram of the measurement points layout and parameters is shown in Fig. 5. The unit virtual force is applied at the target point.

The strain-derived displacement $x_{sd}$ can be expressed as:

20 $$ x_{sd} = \frac{1}{6}\sum\limits_{i = 1}^{3} {\left[ {\frac{{h_{i} }}{{s_{i} }}(2M_{i - 1} \Delta \varepsilon_{i - 1} + M_{i - 1} \Delta \varepsilon_{i} + M_{i} \Delta \varepsilon_{i - 1} + 2M_{i} \Delta \varepsilon_{i} )} \right]} $$

where $h_{i}$ is the length of *i*th segment; $s_{i}$ means the cross-section width of *i*th segment, which is 3.75 mm for the cantilever beam in this paper; $M_{i}$ is the bending moment at the position of sensor *i*; $\Delta \varepsilon_{i}$ denotes the strain difference at the position of sensor *i*. Then $x_{sd}$ and acceleration are used as the state variables to reconstruct the displacement. Please refer to the literature^26^ for the detailed process of the Kalman filtering algorithm.

**Figure 6:**
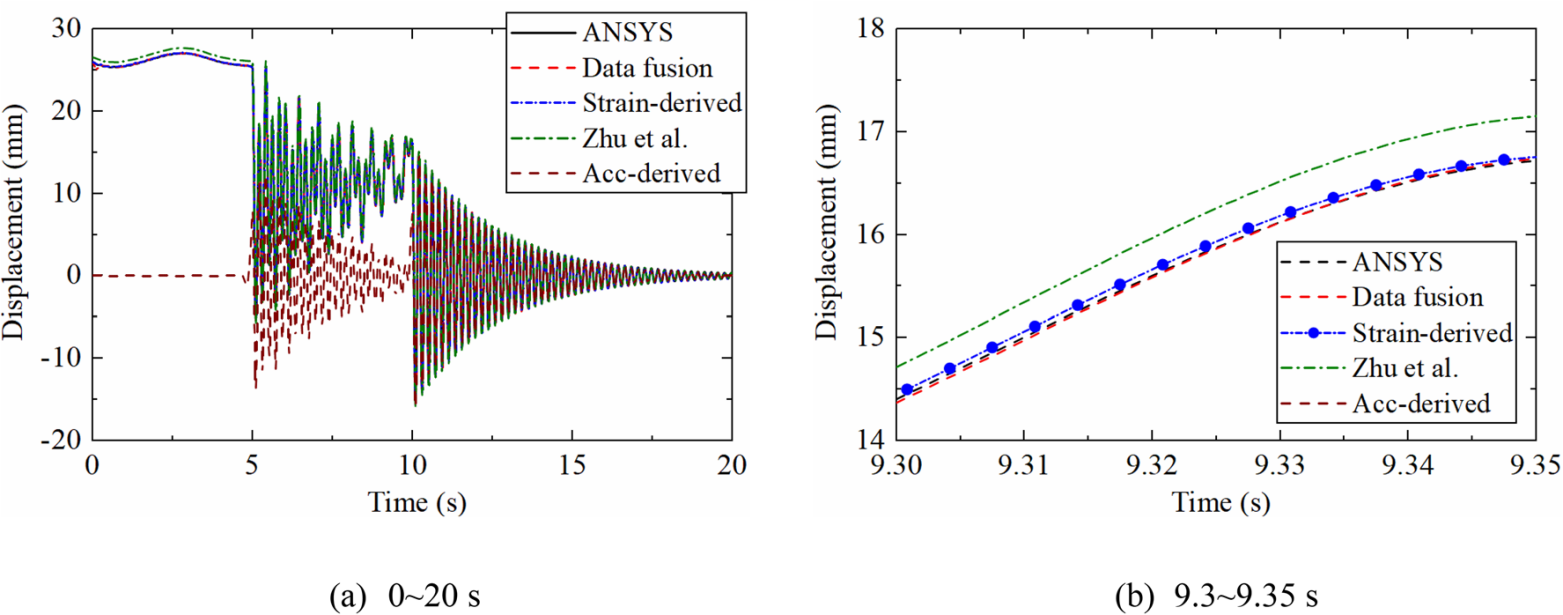
Dynamic displacement reconstructed by different methods.

Figure 6 is a comparison diagram between theoretical displacement time history extracted by ANSYS and the results reconstructed by different methods. The acceleration-based displacement reconstruction method proposed by Lee et al.^15^ and data fusion method proposed by Zhu et al. are used for comparison. In order to verify the correctness of the proposed algorithm, no noise was added to the dynamic response. Figure 6a shows that acceleration-derived displacement has large errors in pseudo-static and non-zero mean displacements. The strain-derived displacement is close to the theoretical value but the sampling rate is lower. The displacement reconstructed by the two data fusion methods are in good agreement with the reference values.

Figure 6b illustrates a partial enlarged view of 9.3–9.35 s. It can be seen that the accuracy of the reconstructed displacement of the proposed algorithm is significantly higher than that proposed by Zhu et al., which proves that the proposed algorithm can still have a good effect even when the number of measurement points is reduced.

To better reflect the difference between forward and backward Kalman filtering algorithms as well as the effectiveness of the fixed interval smoothing technique, Gaussian white noise with a signal to noise ratio of 100 dB was added to the acceleration and strain response. At the same time, the error index $\gamma$ is defined to measure the degree of error between the reconstructed displacement value and the theoretical displacement value:

21 $$ \gamma = \frac{{\sqrt {\frac{1}{N}\sum\limits_{n = 1}^{N} {(d_{r} - d_{t} )^{2} } } }}{{\max (d_{r} )}} \times 100\% $$

where *N* is the total number of data points, $d_{r}$ is the reconstructed displacement value, and $d_{t}$ represents the theoretical displacement value.

**Figure 7:**
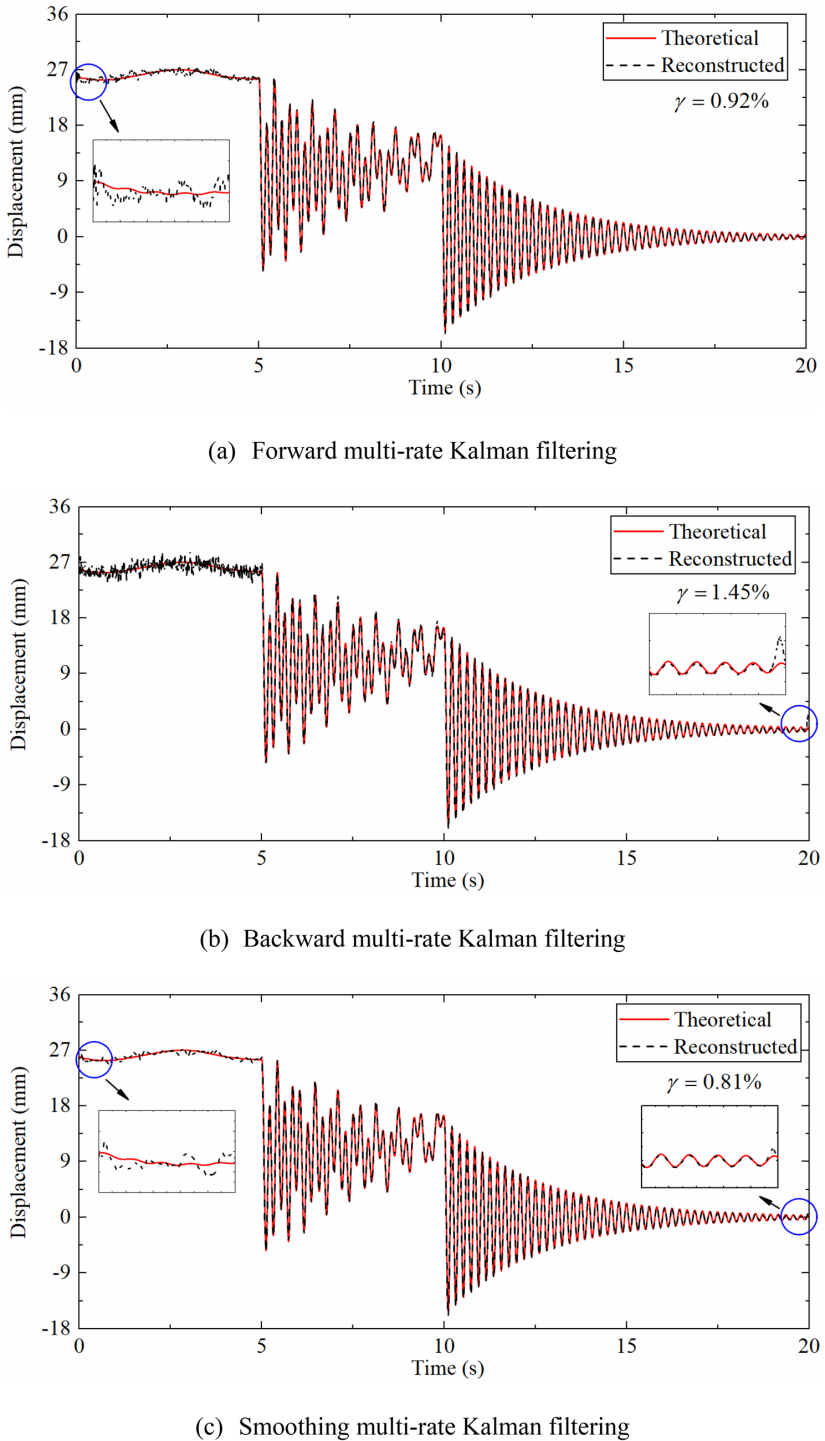
Displacement time histories reconstructed by different multi-rate Kalman filtering algorithms compared with theoretical displacement.

As shown in Fig. 7, the reconstructed displacement curve and the theoretical displacement curve still have a good agreement after adding noise to the dynamic response. Figure 7a,b display that the displacement curves reconstructed by forward Kalman filtering and backward Kalman filtering are not completely consistent. It can be clearly seen that the displacement reconstructed by forward filtering oscillates around 0 s, but the displacement reconstructed by backward filtering oscillates at the end of the time series, namely 20 s. This is because the backward filtering is a reverse operation on the time series, the Kalman gain has a different trend. The reason for the oscillation is due to the inaccurate estimation of the initial value of the velocity, but the displacement error is gradually reduced after the subsequent correction of the algorithm.

Figure 7c illustrates the displacement time history reconstructed by smoothing Kalman filtering. It can be seen that the displacement curve of 0–5 s is obviously smoother. The partial enlargement also shows that the degree of oscillation near 0 s and 20 s is significantly reduced. On the whole, the error indexes of forward filtering, the backward filtering and the smoothing filtering are 0.92%, 1.45%, 0.81% respectively, indicating that the smoothing Kalman filtering technique can improve the accuracy of the reconstructed displacement effectively. The smoothing Kalman filtering algorithm is applied to reconstruct the displacement in the following discussion.

### Parametric analysis

In actual engineering, acceleration and strain response data may have different sampling rates, and noise levels, or reconstructed displacement points may vary. In order to investigate the robustness of the algorithm, three parameters were proposed, namely sampling rate ratio (SRR), signal to noise ratio (SNR), and distance from fixed end (DFF). Among them, the SRR refers to the ratio of acceleration sampling rate and strain sampling rate. Refer to Table 1 for specific working conditions.

**Table 1:** Parameter settings.

Parameter	SRR	SNR (db)	DFF (m)
SRR	4	100	0.8
	20	100	0.8
	50	100	0.8
	100	100	0.8
SNR (db)	4	5	0.8
	4	10	0.8
	4	20	0.8
	4	100	0.8
DFF (m)	4	100	0.2
	4	100	0.4
	4	100	0.6
	4	100	0.8

Acceleration, strain and displacement response data under different working conditions were acquired using ANSYS software. The positions of the applied load point and the strain response extraction point are the same as those in Fig. 2, while the locations of the acceleration and displacement response extraction points were increased by three, which were respectively 0.2 m, 0.4 m and 0.6 m from the fixed end. Then the corresponding white Gaussian noise was added to the acceleration and strain response according to the specific working conditions. For saving space, the follow-up discussion only gives the displacement time history diagram with the largest error in each influencing factor.

**Figure 8:**
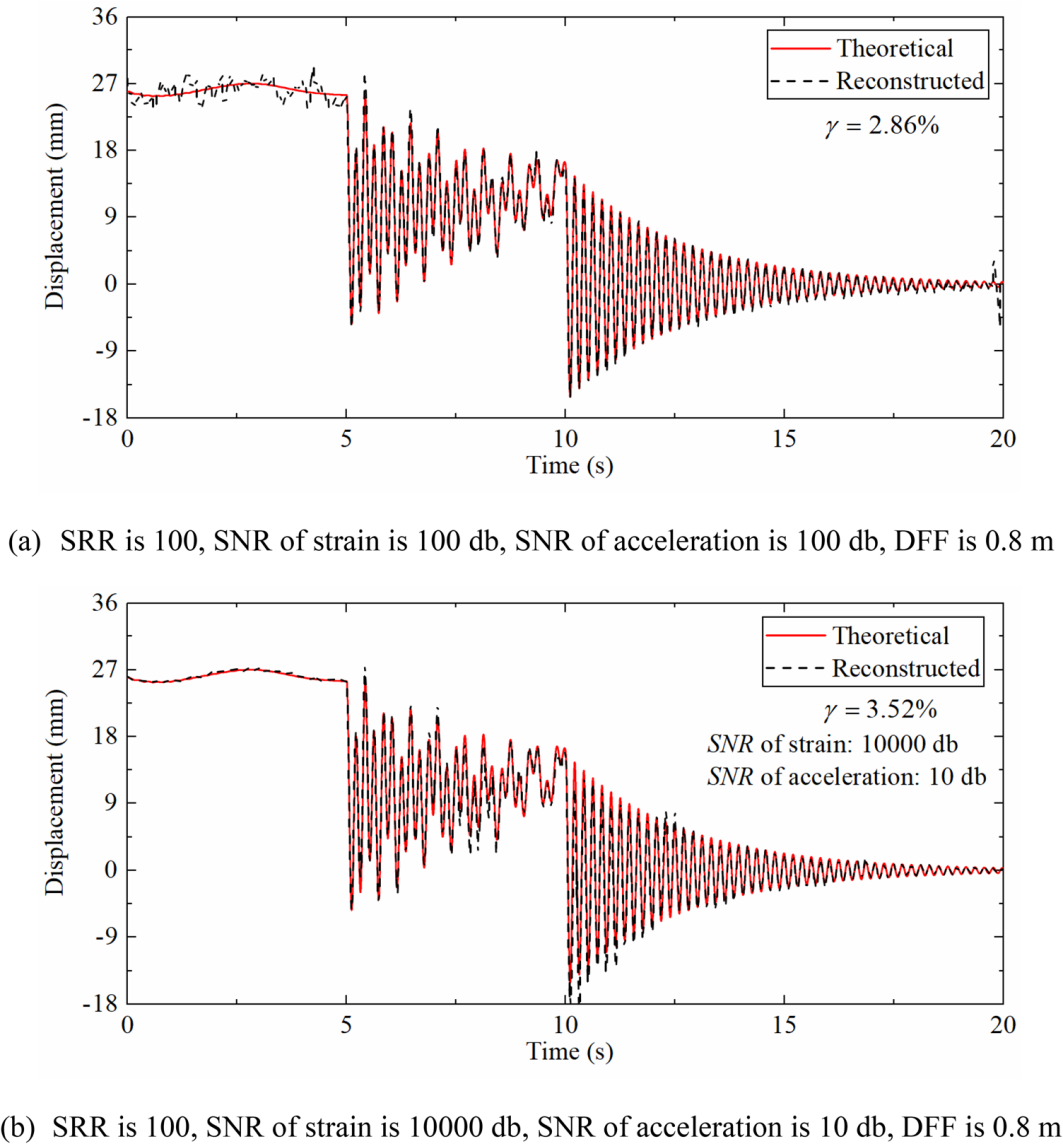
Displacement time history comparison.

Figure 8a displays displacement time history when the SRR is 100 with the SNR of strain and acceleration are the same, it illustrates that the degree of agreement between the two curves is reduced compared with Fig. 7c. This is due to the increase in the SRR results in a longer measurement update interval, which in turn increases the error. The pseudo-static displacement error is larger than that of the high-frequency displacement, but the reduction of the strain sampling rate will not affect the reconstruction of the pseudo-static displacement in fact because of the strain corresponding to the pseudo-static displacement changes slowly. On the contrary, the accuracy of the high-frequency displacement is more limited by the strain sampling rate owing to the rapid change of the corresponding strain. The reason for this situation is that the noise added to acceleration and strain is unreasonable. Figure 8b shows a comparison diagram between reconstructed displacement time history when the SNR of acceleration and strain noise is different and theoretical displacement, which indicates that the pseudo-static displacement error is small and the high-frequency displacement error is large, that confirms to the previous judgment. As a result of the parameter analysis mainly aiming at the robustness of the algorithm, the acceleration and strain noise levels remain consistent in the subsequent discussion.

Table 2 demonstrates the error indexes corresponding to different SRRs. As the ratio of sampling rates increases, the error index $\gamma$ also increases, which is the consequence of the requirement of the strain-derived displacement to guide the update process of the displacement error in the measurement update phase of the Kalman filtering. When the strain sampling rate is reduced, the time interval of the measurement update phase will also become longer and will lead to the larger error of reconstructed displacement. But even when the SRR reaches 100, the $\gamma$ is still only 2.86%, which shows that the proposed data fusion algorithm still has good robustness when the sampling rate ratio is large.

**Table 2:** The error indexes corresponding to the different SRRs.

SRR	4	20	50	100
$\gamma$(%)	0.81	1.35	1.85	2.86

**Figure 9:**
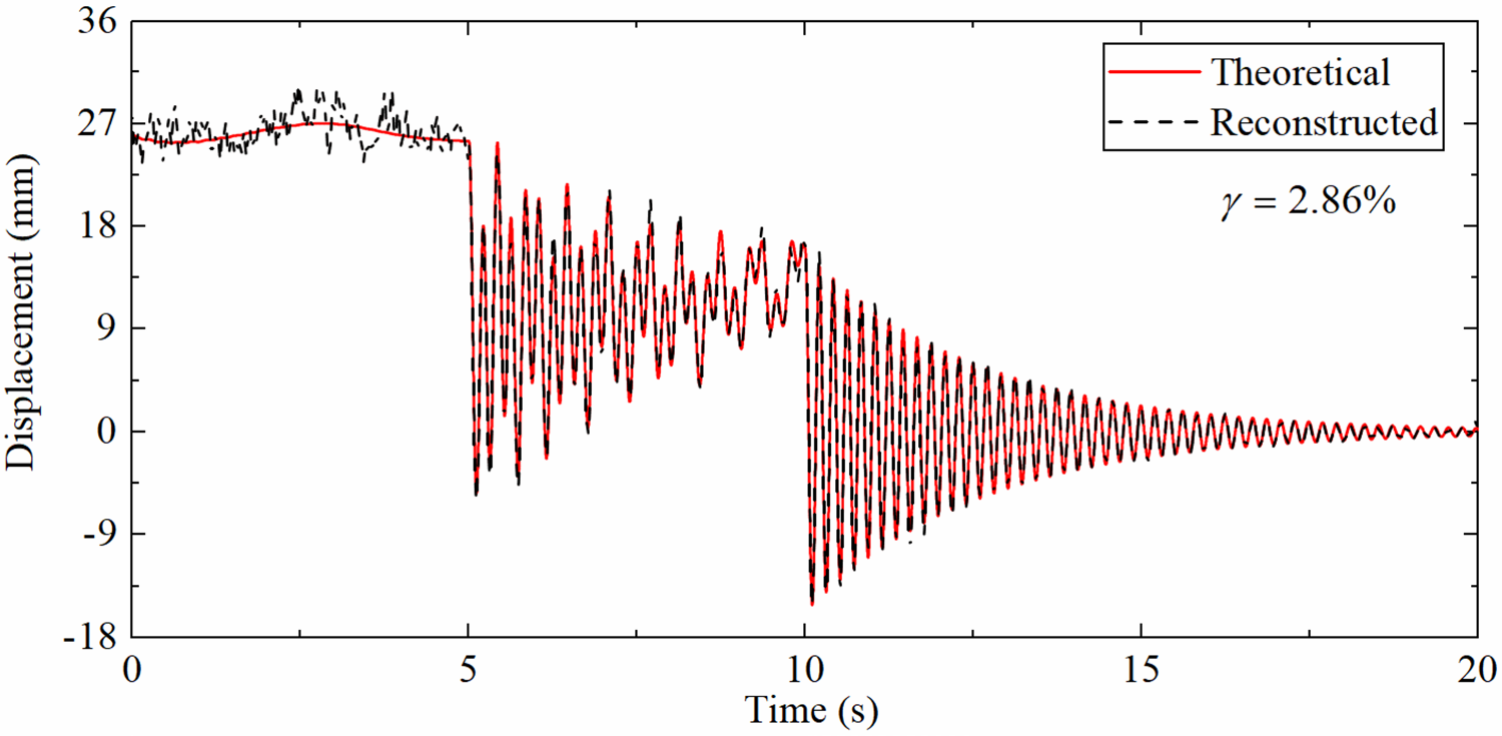
Displacement time history comparison (SRR is 4, SNR is 5 db, DFF is 0.8 m).

As shown in Fig. 9, the accuracy is reduced to a certain extent compared with Fig. 7c, especially in the 0–5 s stage, the pseudo-static reconstructed displacement has been underestimated or overestimated in various degrees at most time points. However, the high-frequency reconstructed displacement within 5–20 s only shows a decrease in accuracy at the peak. Which is since the increase in the SNR makes the strain and acceleration response more inaccurate, yet the acceleration sampling frequency is higher, which can provide more information for the high-frequency displacement reconstruction process, hence, the accuracy change of the high-frequency displacement is smaller than that of the pseudo-static displacement.

Table 3 indicates the error indexes corresponding to different SNRs. It can be seen that as the SNR increases, the error index $\gamma$ gradually decreases. This is because the acceleration and strain responses are closer to the theoretical values as the noise level becomes lower and lower that results the reconstructed displacement being closer to the theoretical values. And when the SNR is reduced to 5 db, the $\gamma$ is only 2.86%, which displays that the proposed algorithm has good anti-noise performance.

**Table 3:** The error indexes corresponding to different SNRs.

SNR (dB)	5	10	20	100
$\gamma$(%)	2.86	2.18	1.68	0.81

**Figure 10:**
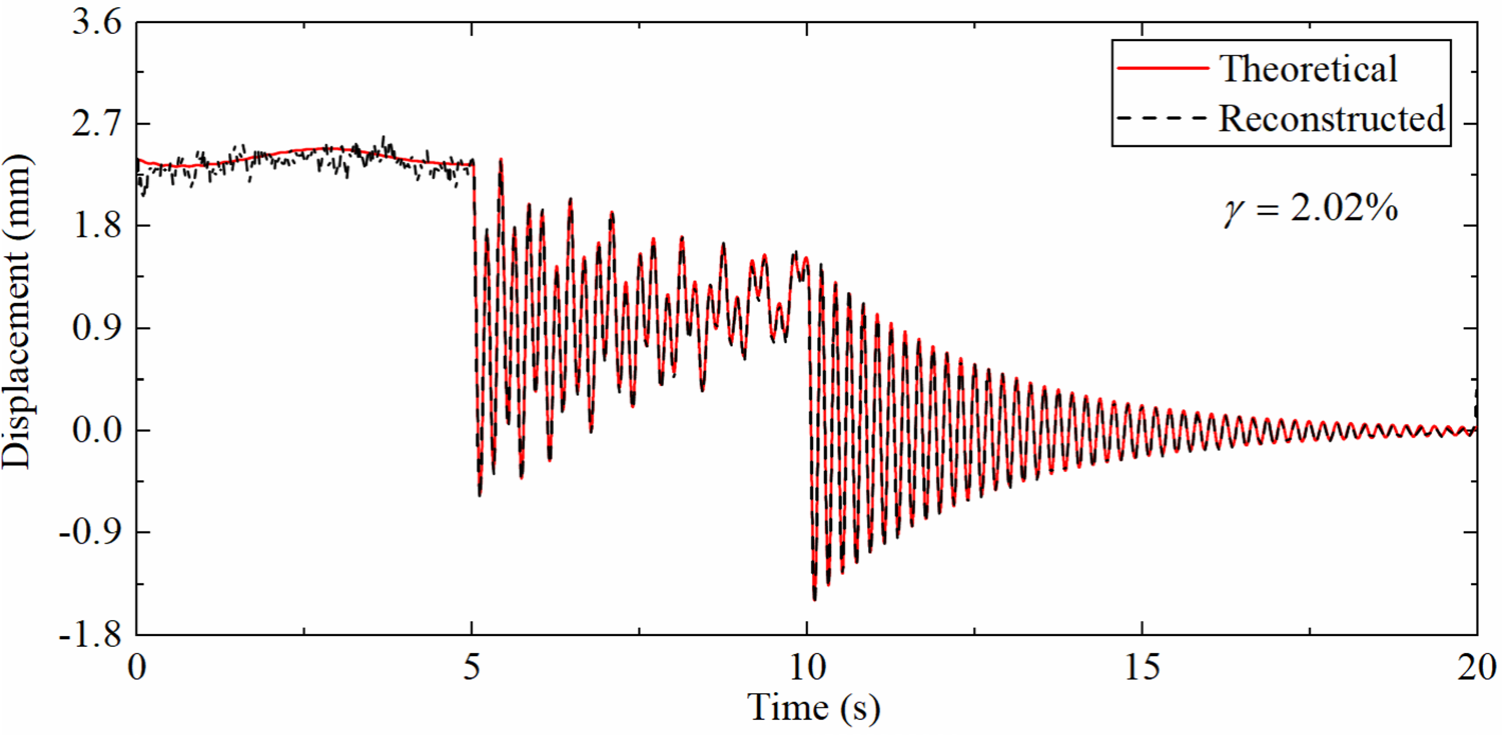
Displacement time history comparison (SRR is 4, SNR is 100 db, DFF is 0.2 m).

Figure 10 describes a comparison diagram between the reconstructed displacement time history at 0.2 m from the fixed end and theoretical displacement. The reconstruction accuracy of pseudo-static displacement and high-frequency displacement is reduced compared with Fig. 7c, which due to the target point is relatively close to the vibration mode zero point of the first two displacement modes of the structure, and the error in obtaining the displacement mode shape from the strain mode shape increases, which leads to a larger strain-derived displacement error.

Table 4 shows the corresponding error indexes at different positions. As the distance from the fixed end increases, the error index $\gamma$ gradually decreases. Which is due to the error of the displacement mode calculated from the strain mode near the fixed end is larger than that of the free end. Therefore, the error of strain-derived displacement is also increased, and the displacement response near the fixed end is smaller, which also makes reconstruction difficult. But the $\gamma$ is only 2.02% when the distance is only 0.2 m, which demonstrates that the proposed data fusion algorithm can effectively reconstruct the displacement closer to the fixed end.

**Table 4:** The error indexes corresponding to different positions.

DFF (m)	0.2	0.4	0.6	0.8
$\gamma$(%)	2.02	1.22	0.94	0.81

## Experimental validation

### Experiment system

To further study the situation when the proposed algorithm is applied to the actual structure, a steel plate was processed into a cantilever beam model for model test. The cantilever beam model is 800 mm long, has a section size of $30 \times 3.75$ mm^2^, an elastic modulus of 210 GPa, and a Poisson's ratio of 0.3. Then it was placed vertically on an optical platform, and five resistance strain gauges are evenly arranged along the height of the beam. The acceleration response was measured by a piezoelectric acceleration sensor, and a laser displacement sensor was used to collect the displacement response of the measurement point. The light emitted by the laser displacement meter, the center point of the strain gauge and the accelerometer should be guaranteed to be on the axis of the cantilever beam, and all sensors should be vertically parallel to the cantilever beam. It is worth noting that the part of the cables close to the structure and sensors are fixed on the structure. Therefore, the cables, sensors and structure can be regarded as one object. This means that the identified mode shape is of this overall structure, and the proposed method will also be applied to this overall structure. Moreover, the strain gauge is fixed on the surface of the cantilever beam by strong glue, the accelerometer is adsorbed on the surface of the cantilever beam through the bottom magnetic seat, and the laser displacement meter is fixed on the other beam opposite to the cantilever beam to be tested by strong double-sided glue.

In order to calibrate all the sensors, the strain gauge and the standard fiber grating strain sensor were installed on both sides of the standard specimen, and use the universal testing machine to load. The sensitivity of the strain gauge is calculated according to the sensitivity of the standard fiber grating sensor. The calibration of the accelerometer is completed by the acceleration sensor calibration system, and the sensitivity of the laser displacement meter can be obtained according to the factory test report of the sensor provided by the manufacturer. The main performance parameters of the sensor are shown in Table 5, it can be seen from the above table that the resolution of the laser displacement meter can reach 0.005 mm, which is enough to ensure the accuracy of the measured value.

**Table 5:** Performance parameters of all sensors.

	Strain gauge	Accelerometer	Laser displacement meter
Measuring range	± 1000 με	50 g	± 500 mm
Resolution	0.02 με	0.0002 g	0.005 mm

**Figure 11:**
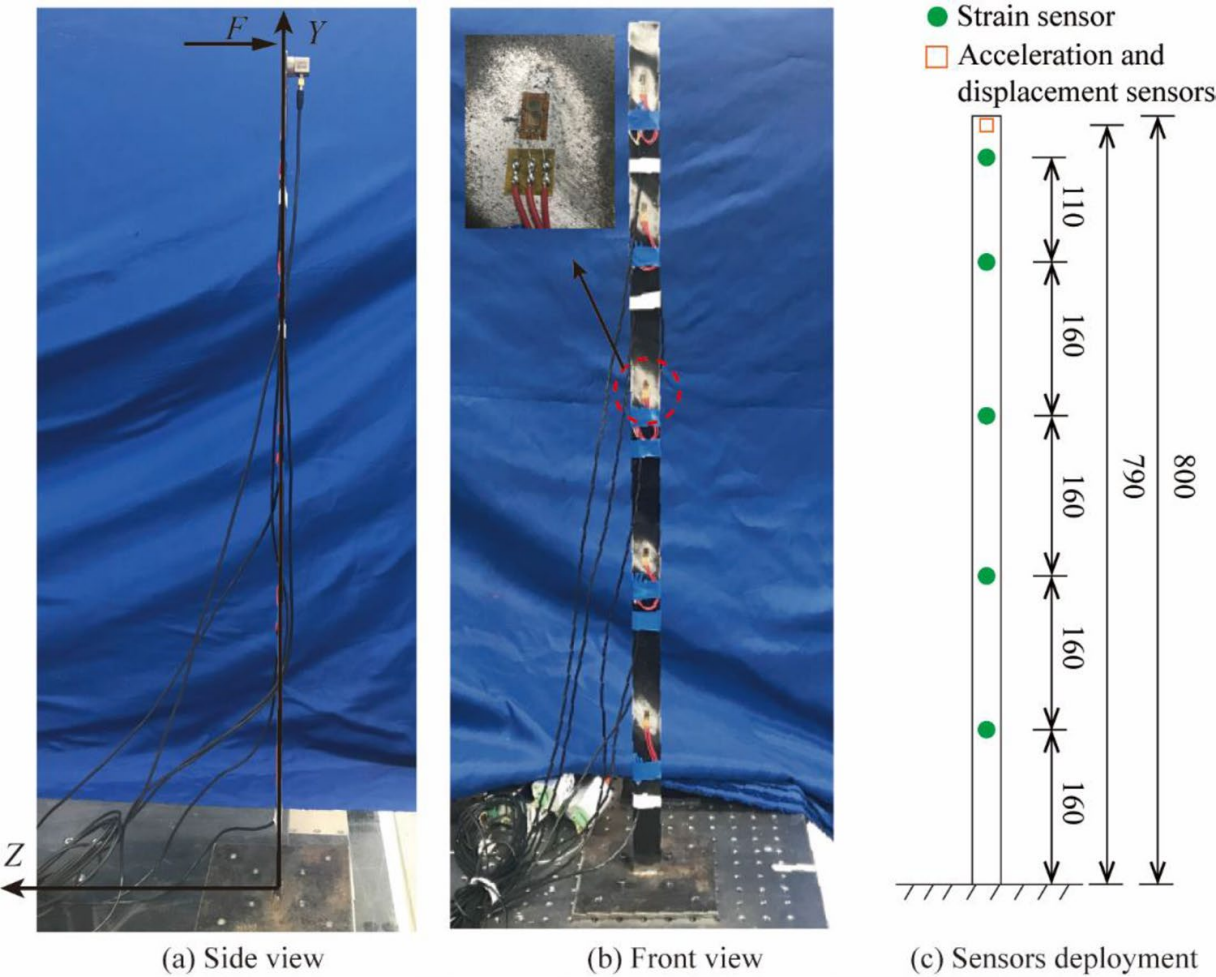
Sensor layout.

The sensor layout is shown in Fig. 11.

**Figure 12:**
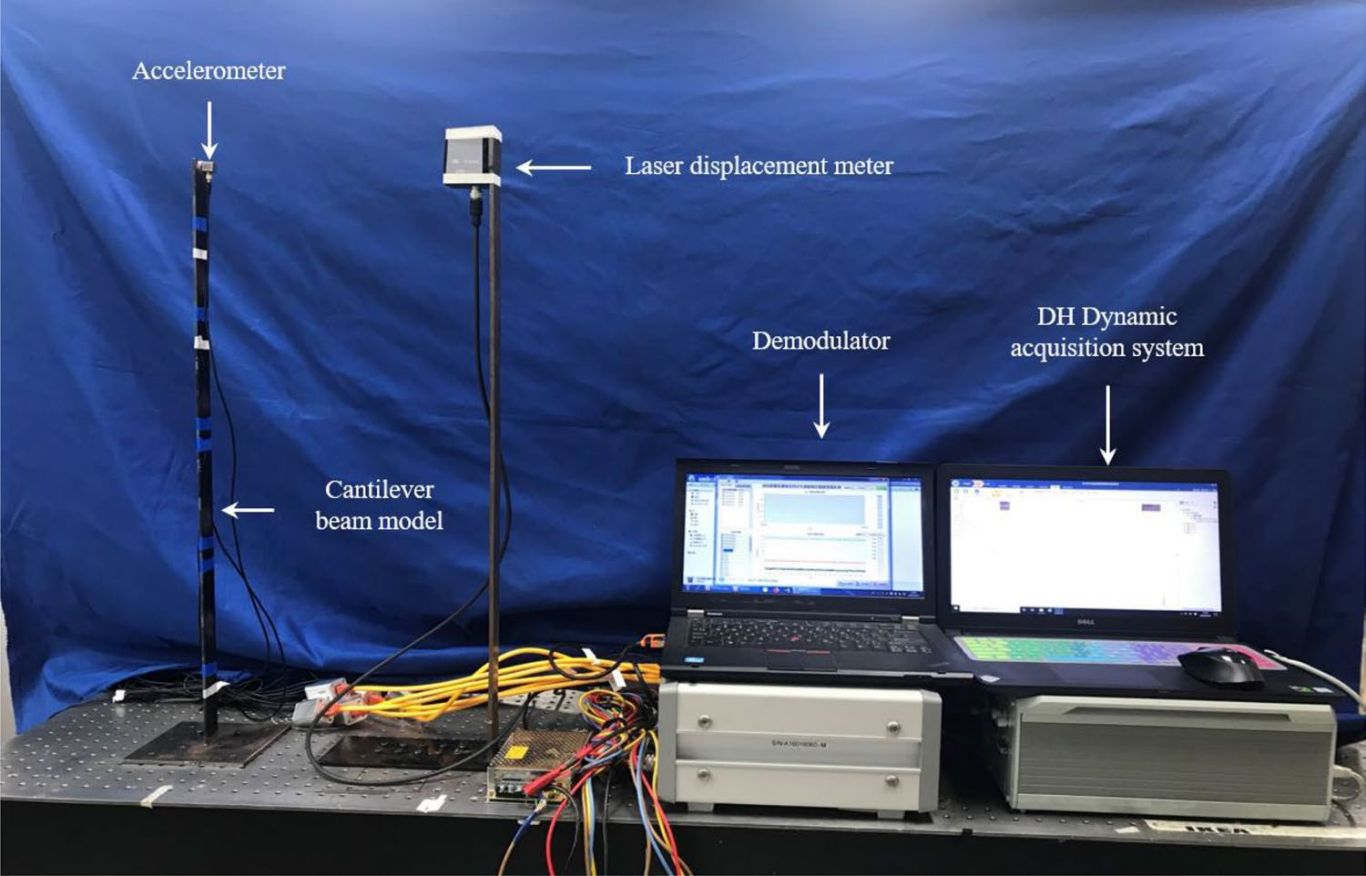
Vibration experiment system.

A coordinate system was also established Fig. 11, and excitation was applied along the *Z* direction. The loading method adopted a manual push method to produce pseudo-static displacement, high-frequency displacement with zero mean and non-zero mean. The acceleration and strain sensors were demodulated by the photoelectric synchronous demodulator independently developed by the research group, and the signal of the laser displacement meter was collected by the Donghua (DH) dynamic signal acquisition and analysis system. The built experimental system is shown in Fig. 12. The acceleration, strain and displacement sampling rate are set to 1200 Hz, 300 Hz and 10 kHz respectively. And the acquisition time was 20 s. The method of resampling was adopted to reduce the displacement sampling rate to 1200 Hz in the later data processing.

### Displacement reconstruction

**Figure 13:**
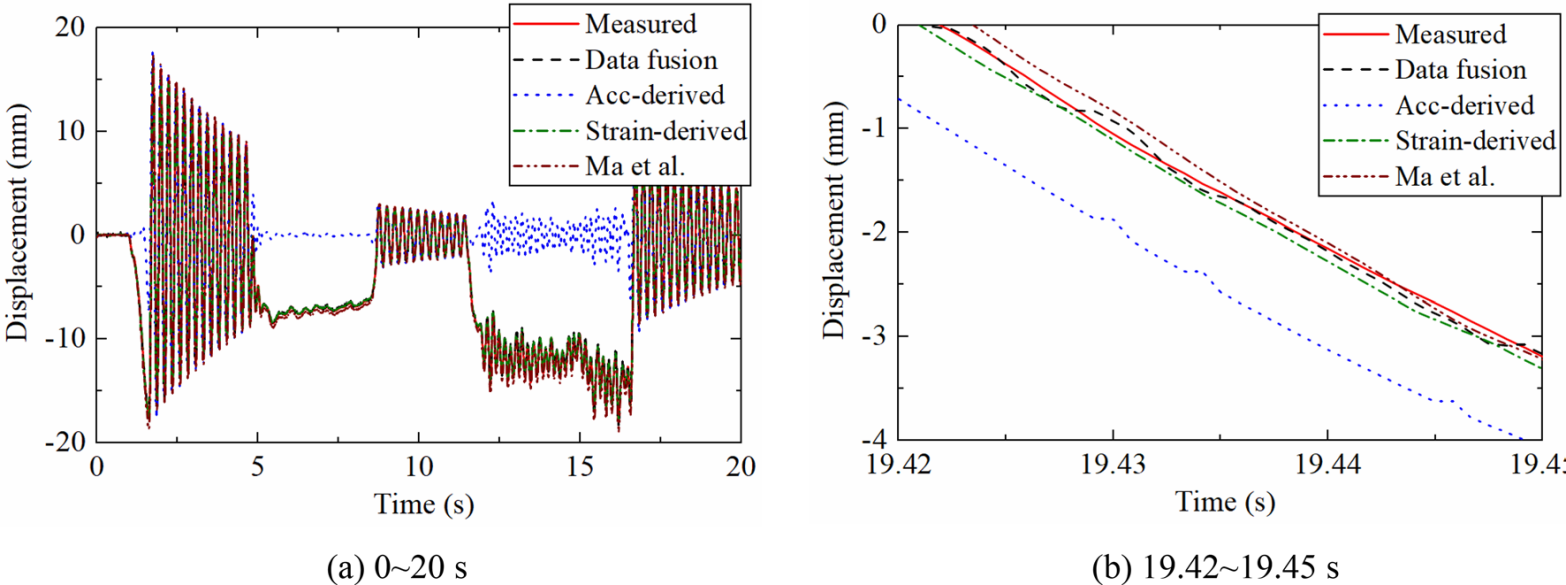
Comparison of displacement reconstructed by different methods.

The data fusion algorithm and SSI method proposed above were used to process the collected dynamic response to realize displacement reconstruction, and the reconstructed displacement was compared with the data collected by the laser displacement meter, the acceleration-derived displacement and the strain-derived displacement. The proposed method was also compared with the existing method proposed by Ma et al.^35^. Figure 13 illustrates that the acceleration-derived results fail to recover non-zero mean and pseudo-static components. The strain-derived displacement and displacement calculated by Ma et al. are very close to the measured values, and the data fusion displacement is the most accurate one, indicating that the reconstruction results are reliable and accurate.

**Figure 14:**
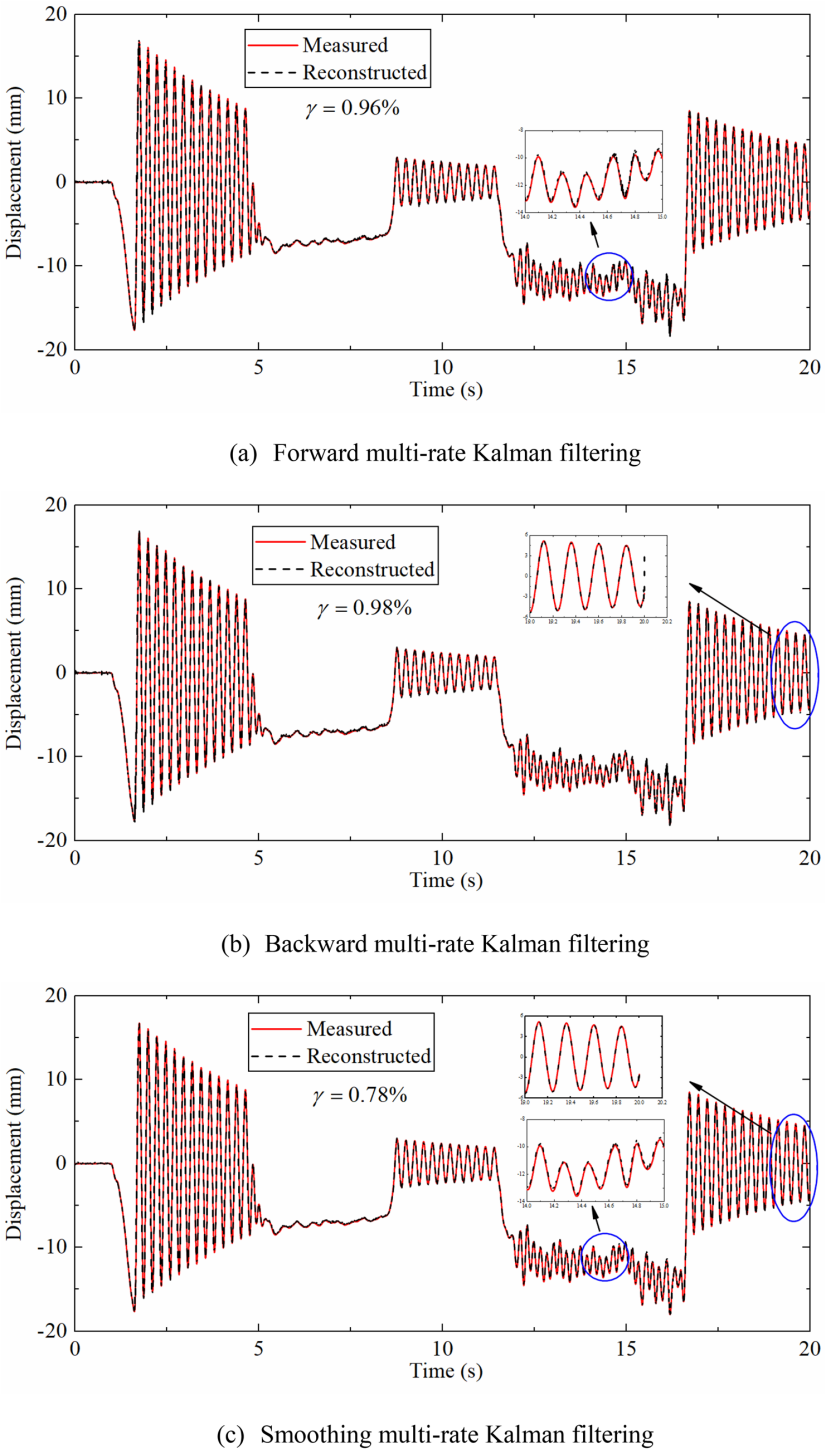
Comparison between displacement reconstructed by different Kalman filtering algorithms and measured displacement.

Different Kalman filter algorithms were used to reconstruct the displacements to show the effectiveness of the smoothing technique. Figure 14a compares the forward filtering displacement and the measured displacement time history of the measurement point at 0.79 m, which can be seen that the reconstructed displacement curve calculated by the data fusion algorithm is in good agreement with the measured displacement. Zero mean high-frequency displacement, non-zero mean high-frequency displacement and pseudo-static displacement are all effectively reconstructed. The error index $\gamma$ is 0.96%, indicating the proposed method has a high reconstruction accuracy.

Figure 14b,c indicates the displacement reconstructed by backward filtering and smoothing filtering and measured displacement respectively. As shown in Fig. 14b, the results of the backward Kalman filtering are not completely consistent with those of the forward. The displacement time history of the backward filtering oscillates around 20 s that does not appear in the forward filtering, which is the same as the numerical simulation section. Figure 14c indicates that the degree of oscillation at the peak is significantly reduced. Overall, the $\gamma$ has reduced from 0.96% to 0.78%, demonstrating that the smoothing Kalman filtering technique can effectively diminish the error, which is consistent with the simulation results in “Case study using a cantilever beam”. The smoothing Kalman filtering algorithm was used to reconstruct the displacement in the following discussion.

### Parametric analysis

Since the actual response collected already contains noise, the influence of noise on the accuracy of the proposed algorithm is no longer analyzed here, and only the influence of SRR and DFF on the accuracy of the algorithm are studied. The specific working conditions are shown in Table 6. The acceleration and displacement sampling rate remain unchanged, and the strain sampling rate changes accordingly with different working conditions.

**Table 6:** Parameter settings.

Parameter	SRR	DFF (m)
SRR	4	0.79
	10	0.79
	20	0.79
	40	0.79
DFF (m)	4	0.2
	4	0.4
	4	0.62
	4	0.79

To study the influence of the distance from the fixed end, it is necessary to arrange the acceleration and displacement sensors to four different measurement points since the laser displacement meter can only measure the dynamic displacement of one point at a time; neither the loading method nor the location of the strain measurement point changes.

**Figure 15:**
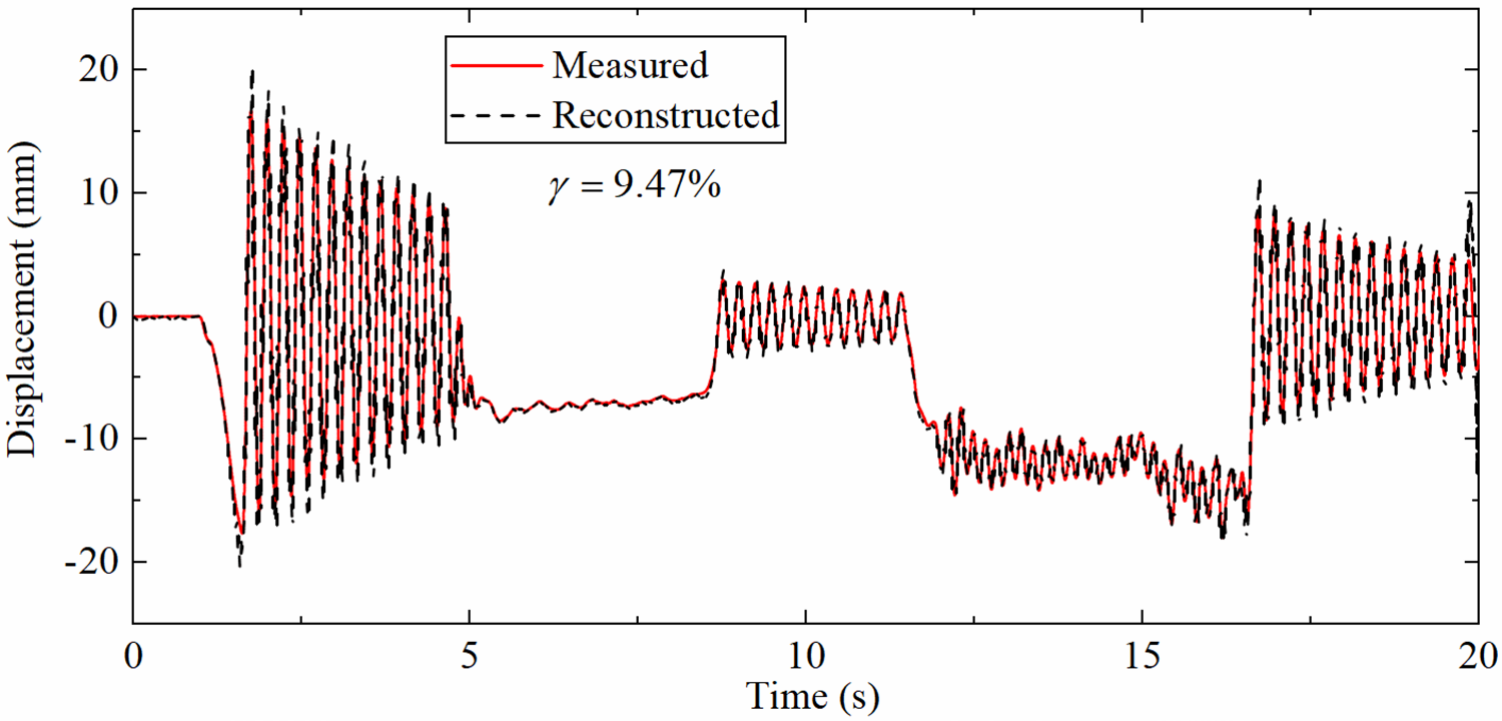
Displacement time history comparison (SRR is 40, DFF is 0.79 m).

As shown in Fig. 15, the accuracy of the reconstructed displacement has been reduced to a certain extent compared with Fig. 14b, and the fitting error of the high-frequency displacement part is larger than that of the pseudo-static displacement. This is due to the pseudo-static displacement has a low change frequency so as the corresponding strain change rate. Even the displacement reconstructed by the strain with a low sampling rate can have a good effect, while high-frequency displacement has a high change frequency, which not only relies on the strain-derived displacement to instruct the measurement update process, but also requires the acceleration integration results to provide information. The decrease of the strain sampling rate makes the measurement update interval longer, which in turn leads to larger high-frequency displacement fitting errors. This also verifies the results of the numerical simulation section.

Table 7 describes the error indexes corresponding to the different SRRs. As the SRR increases, the error index $\gamma$ gradually increases. The above situation also occurs because the increase of the sampling rate makes the measurement update time interval longer, and the fitting error of the high-frequency displacement time history increases. However, when SRR reaches 40, the error index is only 9.47%, illustrating that the algorithm is suitable for situations with low sampling rate strain.

**Table 7:** The error indexes corresponding to the different SRRs.

SRR	4	10	20	40
$\gamma$(%)	0.78	0.98	2.54	9.47

**Figure 16:**
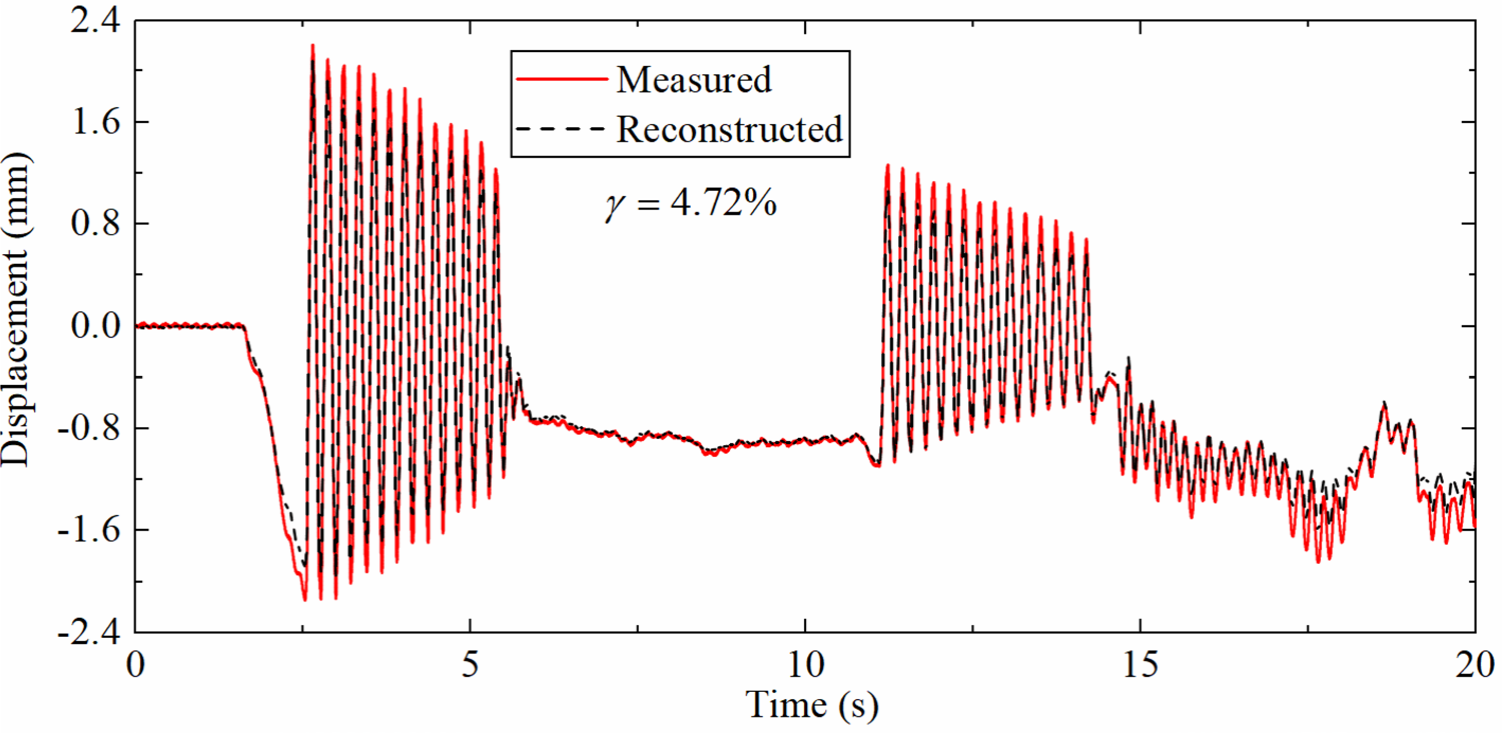
Displacement time history comparison (SRR is 4, DFF is 0.2 m).

Figure 16 displays reconstructed displacement and measured displacement at 0.2 m from the fixed end. It can be found that the accuracy of reconstructed displacement is reduced compared with Fig. 14b. The fitting errors of high-frequency displacements of either zero mean or non-zero mean are larger than those of pseudo-static displacement, which due to the decreasing of signal to noise ratio of acceleration and displacement response at 0.2 m. The reconstructed process of pseudo-static displacement mainly depends on the strain response, and the error is also smaller in comparison.

Table 8 shows error indexes corresponding to different positions. As the DFF increases, the error index $\gamma$ gradually decreases. But not only because the fixed end is located at the mode zero point of the first two displacement modes, but also because the SNR of the acceleration and displacement response near the fixed end decreases, which results in the measurement error increases. The $\gamma$ is 4.72% when the DFF is 0.2 m, which shows that the proposed algorithm can effectively reconstruct the displacement time history of the measurement point near the fixed end in actual measurement.

**Table 8:** The error indexes corresponding to different positions.

DFF (m)	0.2	0.4	0.62	0.79
$\gamma$(%)	4.72	1.35	1.34	0.78

## Summary and conclusion

This paper proposes a method using multi-rate data fusion based on high-frequency acceleration and low-frequency strain responses to solve the problem of displacement reconstruction of beam structure. The SSI method was utilized to process the low sampling rate strain response to calculate the strain mode of the structure, and then derives the low sampling rate displacement response through the mode superposition method; finally, the multi-rate Kalman filtering algorithm is used to correct the acceleration integration error to achieve the purpose of reconstructing the displacement with high sampling rate, which is verified by numerical simulation and cantilever beam model experiment. The proposed method can effectively reconstruct the zero mean, non-zero mean high-frequency displacement and pseudo-static displacement of the target point. Among them, the pseudo-static displacement mainly depends on the accuracy of the strain response measurement, and the high-frequency displacement depends on the measured data of acceleration and strain. The conclusions are as follows:

1. This method has good anti-noise performance. In the numerical simulation, the error index $\gamma$ of reconstructed displacement is only 2.86% when the SNRs of acceleration and strain responses are both 5 db. The dynamic displacement can also be accurately reconstructed using real measurement data with noise in the model experiment.
2. This method can still maintain excellent performance when the acceleration and strain sampling rates differ greatly. In the numerical simulation, the $\gamma$ is 2.86% with a SRR of 100, while the $\gamma$ is only 9.47% when the SRR is 40 in the experiment.
3. For cantilever structures, the closer the position to the fixed end, the greater the error index of reconstructed displacement due to the closer to the displacement mode zero point. In the numerical simulation, the $\gamma$ at 0.8 m from the fixed end is 0.81% meanwhile is only 2.02% at 0.2 m, while the $\gamma$ in the experiment are only 0.78% and 4.72%, respectively, indicating that the displacement reconstruction at a position relatively close to the fixed end also has excellent performance.
4. When the acceleration and strain responses in the entire time series are known, smoothing technology can effectively improve the accuracy of the reconstructed displacement, which is based on the premise of sacrificing the function of real-time online reconstruction of displacement.
5. The proposed data fusion algorithm reconstructs displacements with higher accuracy than those based on strain or acceleration alone.

This method makes full use of high sampling rate acceleration and low sampling rate strain to accurately reconstruct the dynamic displacement of the cantilever beam, and the comparison with existing methods shows that the proposed method has higher accuracy. It is worth noting that this method is also suitable for structures have other boundary conditions (such as simply supported beam) that can use the SSI method to calculate the modal parameters. As a result of this method does not require additional monitoring equipment and only few measurement points are needed, which means that the purpose of real-time dynamic displacement reconstruction with higher precision can be achieved based on the current health monitoring system. The above shows that the proposed method has the advantages of simplicity, economy and real-time efficiency, which is expected to be widely used in the field of structural health monitoring. Although modern DAQ systems can provide synchronized sampling rate for the heterogeneous sensors, there are still many demodulation devices that have been in service for a long time that cannot achieve this. Moreover, the sampling rate of some sensors (e.g. inclinometer) are also limited by their own conditions. To solve this issue, this paper proposes a multi-rate data fusion algorithm, which is applicable to both the same rate and different rate data. This paper conducted a preliminary study on the displacement reconstruction of uniform cross-section beams, but the actual structure usually has a variety of cross-sectional forms. In order to make the proposed method more suitable for actual engineering, the scope of application can be extended to variable cross-section structures in the future.

## Data Availability

All data generated or analyzed during this study are included in this published article [and its supplementary information files].
